# Complete Genome Sequence of *Komagataeibacter hansenii* Strain HUM-1

**DOI:** 10.1128/genomeA.00167-17

**Published:** 2017-04-13

**Authors:** Sarah Pfeffer, Richard Santos, Marcus Ebels, Darius Bordbar, R. Malcolm Brown

**Affiliations:** University of Texas at Austin, Molecular Biosciences, College of Natural Sciences, Austin, Texas, USA

## Abstract

This study reports the release of the complete nucleotide sequence of *Komagataeibacter hansenii* HUM-1, a new efficient producer of cellulose. Elucidation of the genome may provide more information to aid in understanding the genes necessary for cellulose biosynthesis.

## GENOME ANNOUNCEMENT

Organisms contained in the genus *Komagataeibacter* are known to be efficient producers of pure highly crystalline cellulose known as bacterial cellulose (BC) (1 – 3). BC is composed of the same crystalline biopolymer of extended chains of β-1,4-linked glucose residues as other cellulose producers, such as plants, algae, tunicates, protists, and photosynthetic and heterotrophic bacteria (4 – 8). However, unlike those other cellulose producers, *Komagataeibacter* strains employ a hierarchical cell-directed self-assembly process arranged by an evolved genetic code which results in a unique cellulose structure that is ultrafine, highly crystalline, strong, absorbent, moldable, and biocompatible (5, 9 – 12). These characteristics make BC well suited for medical, industrial, and commercial applications (5, 10 – 12). Understanding the mechanisms necessary for the assembly process is crucial for furthering research into this important biopolymer.

This study reports the complete nucleotide sequence of a novel strain of *Komagataeibacter, K. hansenii* HUM-1. This strain was isolated from a hummingbird feeder in Austin, TX, and preliminary observations from our lab have shown it to be an efficient producer of BC. DNA from *K. hansenii* HUM-1 was extracted and subjected to sequencing using an Illumina HiSeq 2000 PE100 system (University of Texas at Austin, ICMB Core Facility). The reads were downloaded into Geneious 8.1.2 and assembled into contigs using Velvet 1.2.02 (13), where it was revealed that the genome is approximately 3.35 Mbp in size, with a G+C content of 59.7% (14). A total of 3,367 open reading frames (ORFs) were predicted using Glimmer (15). Preliminary annotation data on contigs containing cellulose synthase genes were determined.

Phylogenetic analysis using 16S rRNA genes determined that this new strain is closely related to *Komagataeibacter hansenii* ATCC 23769. A homology comparison to the *acsABCD* operon of *K. hansenii* ATCC 23769 (GenBank accession no. AB091060) was performed and resulted in 99.5% identity to *acsAB*, 99.6% identity to *acsC*, and 100% identity to *acsD*. Further investigations into the genome indicated that *K. hansenii* HUM-1 contains a total of three separate coding regions for cellulose biosynthesis: *acsABCD*, *acsAII*, and *acsABC*. These three operons are also found in *K. hansenii* ATCC 23769. A homology comparison of the shared cellulose-synthesizing regions revealed a sequence identity of 76.9% to *acsAII* and 99.6% to *acsABC*. The *acsABCD* operon is flanked by genes coding for proteins which have been determined to be essential for proper cellulose biosynthesis to occur: *cmcAx, ccpAx*, and* bglAx* (16 – 19). The three genes flanking the *acsABCD* operon in *K. hansenii* HUM-1 shared 100%, 99.1%, and 98.9% sequence identity to *K. hansenii* ATCC 23769, respectively.

Further investigations into the genome of *K. hansenii* HUM-1 may provide more insight into the mechanisms necessary for cellulose biosynthesis.

### Accession number(s).

This whole-genome shotgun project has been deposited in DDBJ/ENA/GenBank under the accession no. MJME00000000. The version described in this paper is the first version, MJME01000000.
